# Targeting Oxidative and Immune Dysregulation in Wound Healing via Gold Nanoparticle Conjugate Chitosan

**DOI:** 10.1096/fj.202504913RR

**Published:** 2026-09-25

**Authors:** Sheng‐Der Hsu, Shu‐Jen Chang, Cheng‐An Lin, Gang‐Yi Fan, Hsin‐Da Tsai, Amber Lin, Juin‐Hong Cherng

**Affiliations:** ^1^ Division of Traumatology, Department of Surgery, Tri‐Service General Hospital National Defense Medical University Taipei Taiwan (ROC); ^2^ Division of General Surgery, Department of Surgery Zuoying Armed Forces General Hospital Kaohsiung Taiwan (ROC); ^3^ Department and Graduate Institute of Biology and Anatomy, College of Medicine National Defense Medical University Taipei Taiwan (ROC); ^4^ Laboratory of Stem Cell and Tissue Regeneration National Defense Medical University Taipei Taiwan (ROC); ^5^ Department of Biomedical Engineering Chung Yuan Christian University Taoyuan Taiwan (ROC); ^6^ Department and Graduate Institute of Microbiology and Immunology, College of Biomedical Sciences National Defense Medical University Taipei Taiwan (ROC); ^7^ Graduate Institute of Life Sciences, College of Biomedical Sciences National Defense Medical University Taipei Taiwan (ROC)

**Keywords:** burn trauma, chitosan, gold nanoparticles, immune modulation, oxidative stress

## Abstract

A novel gold nanoparticle‐chitosan (AuNP@chitosan) composite is introduced as a potent biointerface modulator for burn trauma care. In this study, the unique interfacial characteristics of the composite are linked to its therapeutic activity, and its effects on endothelial function and immune response in severe bleeding and inflammatory models are examined. AuNP@chitosan synthesis was verified using FTIR and fluorescence, and its effects were assessed in TNF‐α‐induced HUVECs, LPS‐stimulated macrophages, and an animal model. In vitro, AuNP@chitosan protects TNF‐α‐injured human endothelial cells (HUVECs) by restoring thrombomodulin expression, reducing ROS, and relieving the upregulated hypoxia‐adaptive markers (HIF‐1α and fibronectin). Concurrently, in LPS‐stimulated RAW 264.7 macrophages, it suppresses pro‐inflammatory mediators (TNF‐α and IL‐6) while preserving COX‐1 and enhancing IL‐10 production. These cell‐specific responses reflect distinct but complementary mechanisms, vascular protection and immune regulation, which were further validated in a rat burn model. In vivo application of AuNP@chitosan dressings significantly reduced infiltration of CD4^+^, CD8^+^, and CD68^+^ immune cells in wound tissue, supporting the translational relevance of the dual in vitro findings. The integrated cell and animal studies demonstrate that AuNP@chitosan acts via redox and immune modulation to promote a pro‐healing wound environment.

## Graphical abstract

This gold nanoparticle–chitosan (AuNP@chitosan) composite promotes burn wound healing by simultaneously regulating endothelial and immune responses. In vitro, the composite protects TNF‐α‐injured HUVECs by reducing ROS, restoring thrombomodulin, and upregulating hypoxia‐adaptive markers. Concurrently, it polarizes LPS‐stimulated macrophages by suppressing pro‐inflammatory mediators (PGE_2_, TNF‐α, and IL‐6) while sparing COX‐1 and increasing IL‐10. This dual vascular‐immune mechanism was validated in vivo using a rat burn model, where AuNP@chitosan significantly decreased CD4^+^, CD8^+^, and CD68^+^ immune cell infiltration, establishing its translational therapeutic potential.

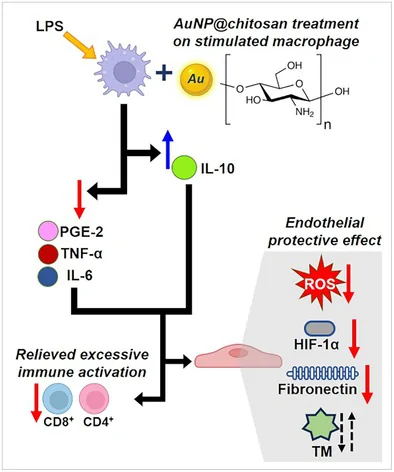

## 1 Introduction

Restoration of vascular homeostasis and inflammation control are critical challenges in the treatment of burn wounds and chronic skin injuries [1]. Endothelial dysfunction, characterized by thrombomodulin (TM) suppression and reactive oxygen species (ROS) accumulation, can amplify coagulation cascades, exacerbate immune cell infiltration, and impair tissue repair processes [2, 3]. The excessive macrophage activation driven by stimuli such as lipopolysaccharide (LPS) sustains chronic inflammation through the release of pro‐inflammatory cytokines, including TNF‐α, IL‐6, and prostaglandin E_2_ (PGE_2_), thereby delaying resolution and epithelial regeneration [4].

Chitosan is a naturally derived polysaccharide that exhibits inherent wound‐healing properties such as hemostatic activity, biocompatibility, and moderate immunomodulation. Incorporation of chitosan in an injured model has demonstrated a shortened hemostasis time and superior antibacterial activity [5, 6]. Its polycationic nature enables it to attract the negatively charged ions in red blood cells and platelets, thus benefiting rapid bleeding control in the initial stage of wound healing [7, 8, 9]. However, its application (e.g., antimicrobial) remains limited owing to reported water solubility issues [10]. Gold nanoparticles (AuNPs) have emerged as a promising strategy. In recent years, AuNPs have garnered considerable attention for use in regenerative medicine owing to their high biocompatibility and relatively low toxicity compared to other nanoparticles. AuNPs are known to scavenge ROS, inhibit NF‐κB activation, and stabilize membrane structures in both endothelial and immune cells [11, 12]. Our group has previously demonstrated the hemostatic and anti‐inflammatory potential of AuNP‐coated chitosan dressings in burn wound contexts [13]. In the present study, AuNPs are surface‐functionalized with dihydrolipoic acid (DHLA)—the reduced, dithiol form of α‐lipoic acid—whose vicinal sulfhydryl groups confer direct reactive oxygen and nitrogen species scavenging capacity. Critically, this DHLA‐mediated antioxidant mechanism operates through direct radical quenching rather than transcriptional upregulation of endogenous antioxidant pathways, providing an immediate, membrane‐proximal ROS suppression that complements the hydrogen‐donor amine groups of chitosan. Together, the DHLA‐functionalized gold core and the chitosan scaffold constitute a chemically defined, dual‐component antioxidant system whose mechanistic basis has not been previously characterized in the context of endothelial coagulation–immune axis regulation. Despite extensive interest in AuNP‐based wound dressings, a major knowledge gap regarding how such nanomaterials coordinately regulate both thrombogenic and inflammatory axes at the cellular and tissue levels persists. Notably, the preponderance of existing AuNP@chitosan wound‐healing research has concentrated on antibacterial performance against multidrug‐resistant pathogens, physical dressing properties, and water solubility enhancement. Neither the restoration of TM—the principal endothelial anticoagulant regulator whose loss defines the coagulopathic phenotype of burn injury—nor the selective modulation of the cyclooxygenase/prostaglandin axis has been reported as a mechanistic endpoint for this composite, representing a critical and unexplored dimension of its therapeutic potential.

Previous research has demonstrated the ability of AuNP‐coated chitosan dressing to shorten hemostasis time and its potential to regulate genes and cytokines related to the wound healing process [13]. In contrast to the prevailing antibacterial and material‐performance paradigm in the AuNP@chitosan field, in this study, we developed and characterized a composite material composed of chitosan integrated with AuNPs (AuNP@chitosan), aiming to evaluate its dual regulatory function in vascular endothelial protection and immune modulation. We specifically positioned endothelial TM loss and macrophage PGE_2_ hyperactivation as the primary pathological targets—a coagulation–immune axis framework not previously reported for this composite. Additionally, surface‐enhanced infrared absorption (SEIRA) spectroscopy was employed to mechanistically link the composite's gold‐core interfacial chemistry to its biological activity, an approach absent from prior AuNP@chitosan characterization studies. Using TNF‐α‐injured human umbilical vein endothelial cells (HUVECs), we assessed the impact of AuNP@chitosan on TM expression, oxidative stress, and hypoxia‐responsive genes such as HIF‐1α, fibronectin, and TAF‐1. Concurrently, we investigated its immunological effects in LPS‐activated RAW 264.7 macrophages, focusing on key inflammatory mediators (TNF‐α, IL‐6, and IL‐10), PGE_2_, and cyclooxygenase isoforms, including COX‐1. The in vitro findings were further validated in a rat burn wound model. Immune cell infiltration (CD4^+^, CD8^+^, and CD68^+^) was quantified to assess in vivo immunomodulatory effects.

By bridging endothelial and immune mechanisms with translational animal data, this study aims to establish AuNP@chitosan as a next‐generation biointerface material capable of restoring coagulation balance and resolving inflammation in wound healing contexts. The elucidation of selective ROS suppression, TM recovery, and cytokine reprogramming provides novel insights into nanomaterial‐mediated immune‐endothelial crosstalk and its therapeutic implications.

## 2 Materials and Methods

### 2.1 Preparation and Interfacial Characterization of AuNP@Chitosan

Water‐soluble AuNPs@DHLA were synthesized using the etching method described by Lin et al. [14]. Briefly, 100 mM DDAB and 25 mM AuCl_3_ were prepared in toluene. TBAB and the gold precursor solution were added to a decanoic acid solution to obtain 6‐nm AuNPs. Lipoic acid was added (4:1 molar ratio to TBAB), and the cells were exposed to 365 nm UV light for 20–30 min. The AuNPs@DHLA concentration was adjusted to 40 μM after purification (100 and 30 kDa MWCO filters).

The chitosan raw material (Formula: [C_8_H_13_NO_5_] *n*; 100 kDa, 85% degree of deacetylation) was purchased from Une Shin Trading Co. Ltd. (CAS No. 9012‐76‐4; New Taipei City, Taiwan, ROC), dissolved in 1% acetic acid (0.5% concentration), and stirred at 25°C. EDC was used as a crosslinking agent with 40 nm COOH‐AuNPs. For bioconjugation, 10 μL AuNPs@DHLA (40 μM), 10 μL chitosan 1 mM, and 10 μL EDC dilutions (256 × 2^−*n*^ mM; *n* = 0–11) were mixed. FTIR and UV–VIS spectroscopy were used to verify the successful formation of the composite and to examine the chemical nature of its surface, which is crucial for its biointerfacial activity. Samples were analyzed using 2% agarose gel electrophoresis (40 min, 7.5 V cm^−1^, Bio‐Rad), and AuNP@chitosan conjugates were observed under UV light (365 nm).

The concentration of the AuNP@chitosan wound dressing was 20 μM AuNP with 0.5% (w/w) chitosan. Further dilutions of AuNP@chitosan to 1/20, 1/50, 1/100, and 1/200 were also prepared for treatment. All concentration–response experiments were evaluated at fixed end points (time‐independent analysis: 18–24 h posttreatment for in vitro assays; Day 7 post‐injury for in vivo assays).

### 2.2 Cell Culture and TNF‐α Injury Model

Human umbilical vein/vascular endothelial cells (HUVECs, BCRC: H UV001) were cultured in F‐12 K medium (ATCC 30‐2004) (37°C, 5% CO_2_) supplemented with 10% FBS and 1 U/mL streptomycin–penicillin. TNF‐α stimulation (20 ng) mimics the injured endothelium condition during severe bleeding. Treatments (AuNPs, chitosan, and AuNP@chitosan) were applied to the model (cells with and without TNF‐α stimulation served as controls).

The murine macrophage cell line RAW 264.7 (ATCC, TIB71, Rockville, MD, USA) was grown in Dulbecco's modified Eagle's medium (37°C with 5% CO_2_) without phenol red, supplemented with 10% fetal calf serum, 4 mM d‐glutamine, penicillin (100 U/mL), and streptomycin (100 μg/mL). Evaluation after AuNP@chitosan treatment (various concentrations, 18 h) was conducted after LPS stimulation (200 ng/mL, 6 h).

### 2.3 Cell Viability and ROS Measurement

HUVECs and RAW 264.7 cells (5 × 10^3^ cells/well) (*n* = 3) were cultured in 96‐well plates. For cytotoxicity, CCK‐8 solution (10 μL) was added (2 h, 37°C), and the absorbance was measured at 450 nm. For ROS measurement, cells were incubated with 5 μM CellROX Green reagent (37°C, 30 min), then fixed with 3.7% formaldehyde, and fluorescence was measured using a SpextraMax M3 microplate reader (excitation: 485 nm; emission: 520 nm).

### 2.4 Hemostasis, Coagulation Factor, and Platelet Aggregation Analysis

The indicated proteins were measured according to the manufacturer's instructions (*n* = 3): HMGB and TM (BioVision ELISA Kit; BioVision Inc., CA, USA) and PGE_2_, TNF‐α, IL‐6, and IL‐10 (BioSource International, CA, USA). Briefly, cell culture supernatants (500 μL) from each group were centrifuged (2000 × *g* for 5 min). Cytokine concentrations were determined using a standard curve of known concentrations (samples are kit‐provided). Cells without TNF‐α or LPS treatment were used as controls.

### 2.5 Real‐Time PCR

Total RNA was extracted using the AllPrep DNA/RNA Mini Kit (Qiagen, Hilden, Germany), reverse‐transcribed using PrimeScript RT Master Mix (TaKaRa, Japan), and amplified using a PCR System 9700 thermal cycler (GeneAmp, ABi, Carlsbad, CA, USA). Real‐time PCR of relative gene levels (H1F1‐α, TM, TAF1, fibronectin, HMGB1, COX‐1, PGE‐2, and GAPDH) was performed using SYBR Premix Ex TaqTM (TaKaRa, Japan) and the CFX96TM real‐time system (Bio‐Rad, Hercules, CA, USA) according to the manufacturer's protocol. The experiments were conducted in triplicate.

### 2.6 Animal Burn Injury

All procedures involving animals were reviewed and approved by the Institutional Animal Care and Use Committee (IACUC no. 24‐058, National Defense Medical University). A total of 64 male Sprague–Dawley rats (3 months of age, 250–300 g; BioLASCO Co. Ltd., Taipei, Taiwan) were randomly divided into four groups (*n* = 16): control, untreated burn injury, injury with chitosan treatment, and injury with combined AuNP and chitosan treatment (AuNP@chitosan). After a 7‐day acclimation period, the rats were fasted overnight. On Day 8, the rats were sedated with Zoletil 50 (20 mg/kg, i.m.; Verbac, France). The dorsal fur was shaved (except for the control group), and the skin was disinfected with povidone‐iodine solution. A full‐thickness, third‐degree scald burn was inflicted by applying a 190°C brass block to the dorsal skin for 20 s. The wounds were dressed, covered, and disinfected daily with iodine solution. Seven days post‐injury, the rats were anesthetized with ketamine and xylazine (100 and 10 mg/kg, i.p.) and euthanized by CO_2_ inhalation. The skin surrounding the burn wound was harvested, flash‐frozen in liquid nitrogen, and stored at −80°C. Tissues were rinsed with phosphate‐buffered saline and fixed in 4% paraformaldehyde solution.

A porcine contact thermal injury model was utilized to establish a clinical benchmark for silver‐based treatment [15]. Briefly, 15 pigs were acclimated on a standard diet, fasted overnight, and managed for pain using transdermal fentanyl patches (50 μg/h). Before burn induction, animals were sedated with Zoletil 50 (25 mg/kg, IM), endotracheally intubated, and maintained under anesthesia using isoflurane (0.5%–2.5%). Vital signs were monitored continuously. Following shaving and povidone‐iodine preparation, third‐degree burns were induced on the dorsal flank by applying a brass block (5 cm diameter, 100°C) for 20 s.

To distribute treatments evenly, the 15 animals were randomized into three parallel dressing groups (*n* = 5 per group); for the purpose of this clinical benchmark comparison, data from the untreated standard gauze control group, the calcium alginate dressing (CAPS) group, and the commercial AQUACEL Ag group (*n* = 5) are presented. Values were expressed as percentages relative to the untreated standard gauze control group to evaluate relative changes between CAPS and AQUACEL Ag treatments (Figure S2). Sixty minutes post‐injury, wounds were dressed, secured with nonstick pads, adhesive dressings, and protective garments. Postoperative fluid resuscitation was provided via intravenous saline (0.1 L/kg). For systemic cytokine evaluation, serum samples were collected at Days 0, 1, and 7, and the levels of IL‐4, IL‐6, IFN‐γ, MCP‐1, TNF‐α, TGF‐β1, TGF‐β2, and TGF‐β3 were quantified using a magnetic bead‐based multiplex assay (MILLIPLEX MAP, Merck KGaA) on an xMAP instrument. Similarly, an independent burn experiment was conducted in rats using these same dressing groups (*n* = 2 biological replicates per group; the gauze group represents individual measurements). Serum was collected on Days 0, 1, 3, 7, 14, and 28 to analyze IL‐6 and TNF‐α levels via the same multiplex system (Figure S1).

### 2.7 Histology Study

Immunohistochemical (IHC; CD4: Santacruz #sc‐13573; CD8: SYSY #361003; CD68: abcam #ab125212) staining and immunofluorescence (IF; HIF‐1α: abcam #179483) were performed on harvested burned skin tissue according to the manufacturer's guidelines. The tissues were observed and photographed under a ZEISS LSM 800 upright microscope at 100× magnification for IHC and 10× magnification for IF, and both groups were semiquantitatively analyzed (ImageJ software). Semi‐quantitative analysis of IF images was performed using random square regions per image, selected for their similar measured values. Statistical significance was assessed using the Student's *t*‐test.

### 2.8 Ethics Approval and Consent to Participate

HUVECs (BCRC: HUV001, Taiwan) and RAW 264.7 macrophages (ATCC, USA) were used in accordance with institutional guidelines. The animal experimental protocols were reviewed and approved by the authors' IACUC and complied with the ARRIVE guidelines.

### 2.9 Statistical Analysis

All collected data were analyzed using SPSS software (version 18.0; SPSS, Chicago, IL, USA) and are presented as the mean ± standard error of the mean. Wound‐healing progression among the treatment groups was evaluated using two‐way analysis of variance (ANOVA) with Dunnett's post hoc test, using the lesion group as a reference. Gene expression data from qPCR were assessed using either one‐way ANOVA or paired Student's *t*‐test (two‐tailed). Statistical significance was set at *p <* 0.05.

## 3 Results

### 3.1 Verification of AuNP@Chitosan

**FIGURE 1:**
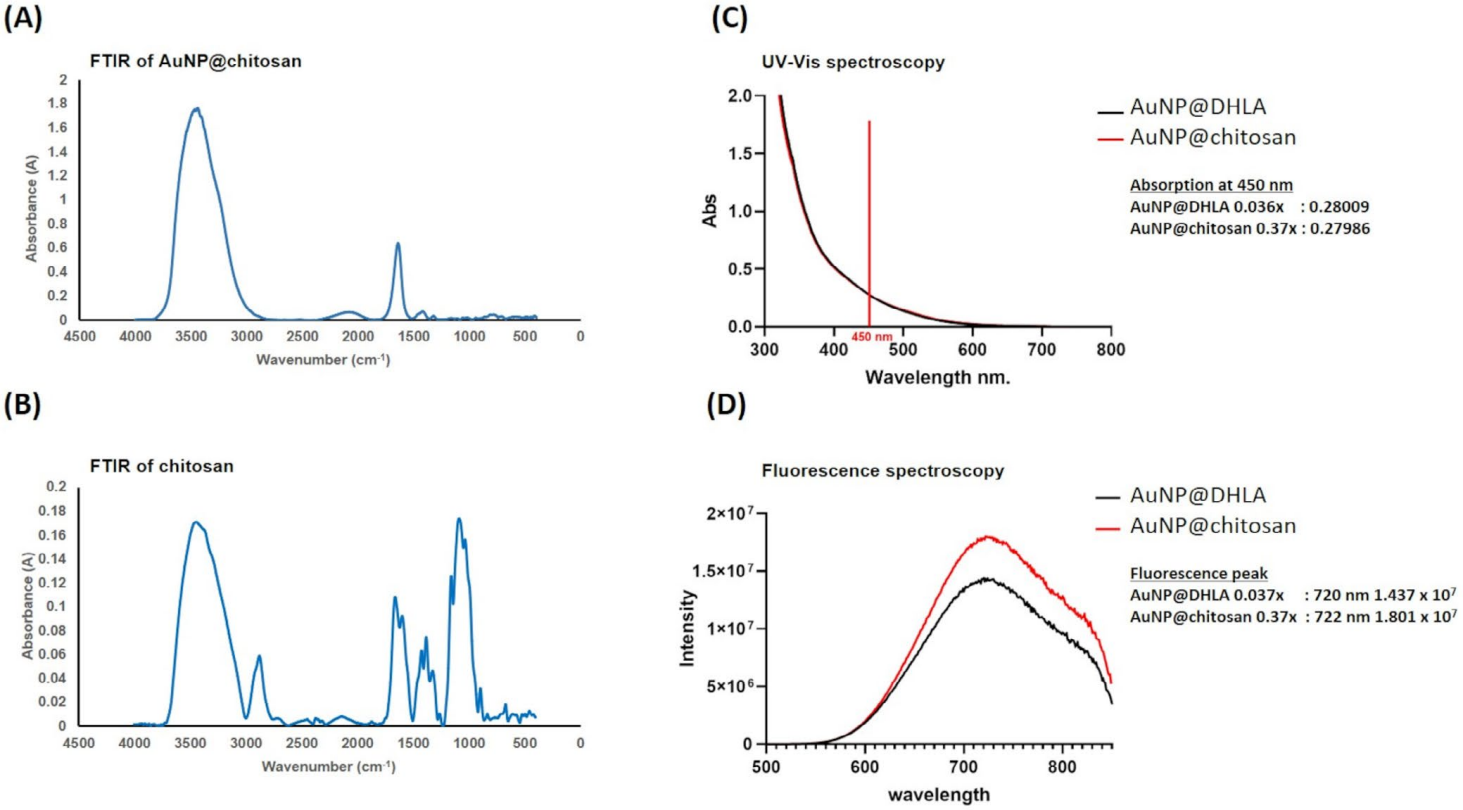
Spectral characterization of chitosan and AuNP@chitosan by FTIR, UV–Vis, and fluorescence emission spectroscopy. (A) FTIR spectra show characteristic peaks of chitosan at 3423 cm^−1^ (O–H/N–H stretching), 1652 cm^−1^ (Amide I), and 1595 cm^−1^ (Amide II). Upon gold nanoparticle incorporation, (B) AuNP@chitosan exhibits spectral shifts, particularly a reduction in intensity at 1652 and 3423 cm^−1^, indicating AuNP interaction with amino and hydroxyl groups. (C) UV–Vis spectra reveal a distinct surface plasmon resonance peak at ~450 nm in AuNP@chitosan, confirming the presence of gold nanoparticles, while native chitosan lacks this absorbance band. (D) AuNP and AuNP@chitosan exhibit broad fluorescence emission peaks centered around 720 and 722 nm, respectively. It is primarily attributed to the intrinsic fluorescence of its amino groups.

First, FTIR spectroscopy was used to verify the successful coating of AuNPs with chitosan and to understand the composite's surface chemistry. A comparative analysis of the AuNP@chitosan composite and pure chitosan revealed a distinct shift and broadening of the Amide II band (~1590 cm^−1^). This confirms a direct chemical interaction between the nanoparticles and amine functional groups of the polymer, which is a key interfacial phenomenon. This interaction was further supported by a notable increase in the absorbance intensity, an effect attributed to SEIRA from the gold core [14]. Collectively, the FTIR data provide clear evidence for the stable formation of the nanocomposite (Figure 1).

After structural verification, the optical properties of the nanoparticles were investigated to confirm their composite nature. AuNPs coated with DHLA and chitosan (AuNP@chitosan) exhibited enhanced fluorescence compared to those stabilized with only DHLA (AuNP@DHLA). While both samples absorbed light at 450 nm, the AuNP@chitosan conjugate produced a fluorescence peak at 722 nm with a considerably higher intensity (1.801 × 10^7^) than the AuNP@DHLA peak at 720 nm (1.437 × 10^7^) (Figure 1). Together, these analyses confirmed the successful synthesis and functionalization of the AuNP@chitosan composite.

### 3.2 AuNP@Chitosan Modulates the Endothelial Biointerface to Enhance Cell Viability and Reduce Oxidative Stress

**FIGURE 2:**
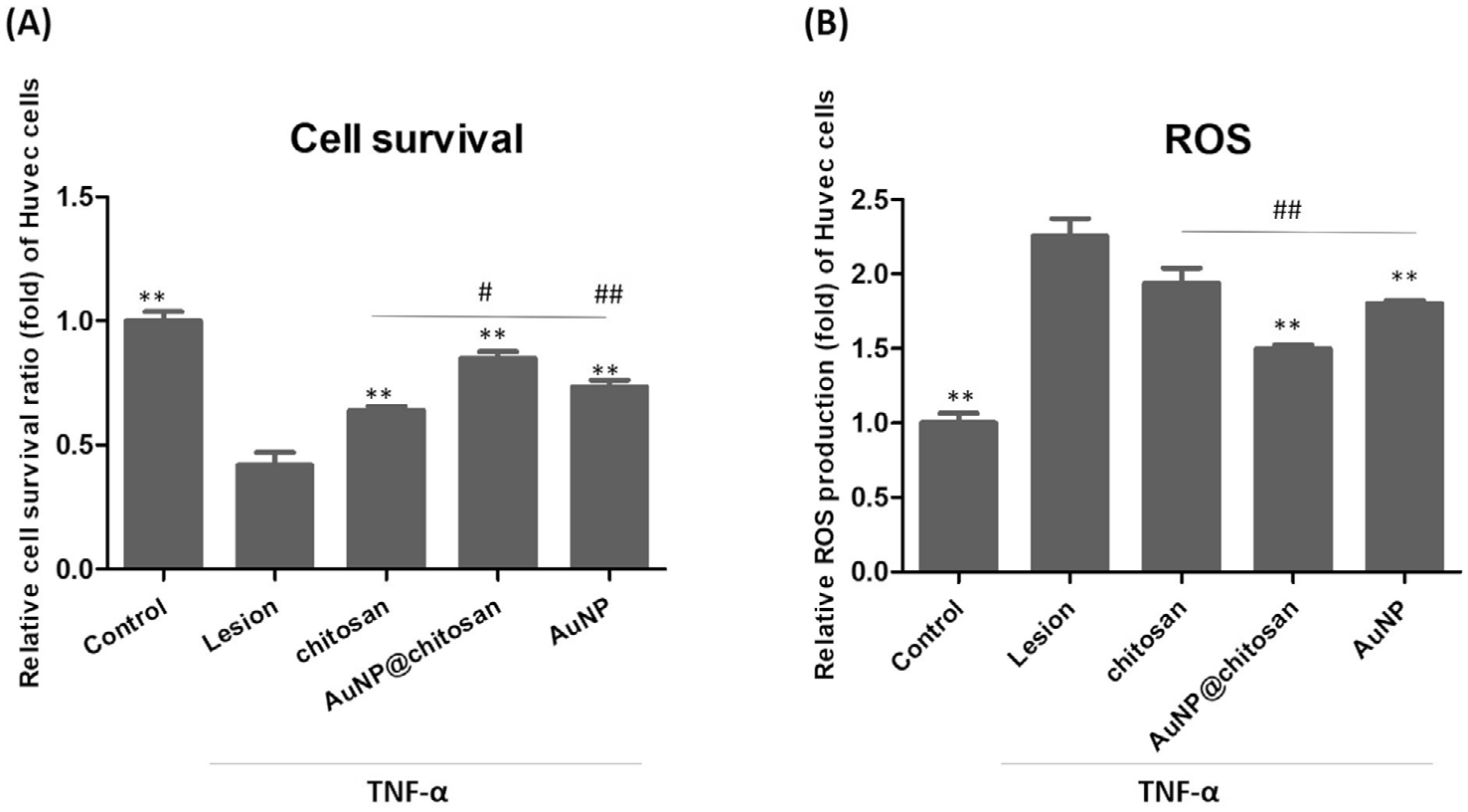
Intracellular ROS generation by CellROX Green in HUVECs under TNF‐α stimulation and treatment conditions. (A and B) ROS fluorescence intensity measured in HUVECs showed that TNF‐α markedly elevated intracellular ROS levels (2.25 ± 0.12‐fold vs. control). Treatment with AuNP@chitosan significantly reduced ROS accumulation to 1.50 ± 0.02‐fold, outperforming both chitosan alone and AuNPs alone, consistent with the direct‐scavenging mechanism described in Section 4 (**p < 0.01, compared with lesion; ^#^ *p* < 0.05, ^##^ *p* < 0.01, compared with chitosan).

The HUVECs serve as a representative model for assessing endothelial functionality, TM expression, oxidative stress response, and vascular behavior [9]. The cells were exposed to TNF‐α, which significantly decreased their viability to 0.41 ± 0.05‐fold relative to the control. Both the individual chitosan and AuNP treatments improved cell viability by 0.64 ± 0.03‐fold and 0.73 ± 0.02‐fold, respectively. However, AuNP@chitosan treatment was more effective, increasing cell viability to 0.85 ± 0.03‐fold (Figure 2), suggesting a potential additive or synergistic effect of AuNPs.

ROS levels, a key cause of oxidative stress and cell death [16, 17], were also measured. TNF‐α treatment increased ROS levels by 2.25 ± 0.12‐fold compared to the control. Individual AuNP treatment reduced ROS levels to 1.80 ± 0.02‐fold of the control; meanwhile, AuNP@chitosan treatment lowered them even further to 1.50 ± 0.02‐fold (Figure 2). Chitosan had a less pronounced effect on ROS levels. Although chitosan and AuNPs individually may reduce ROS formation and improve endothelial cell survival and proliferation [16, 17, 18], their combination has shown greater promise in reducing ROS production in injured endothelial cell microenvironments. This superior effect is a direct result of the unique biointerface presented by the composite, which facilitates a more potent interaction with the cell membrane.

### 3.3 AuNP@Chitosan Mitigates Endothelial Dysfunction by Modulating Key Interfacial Proteins

**FIGURE 3:**
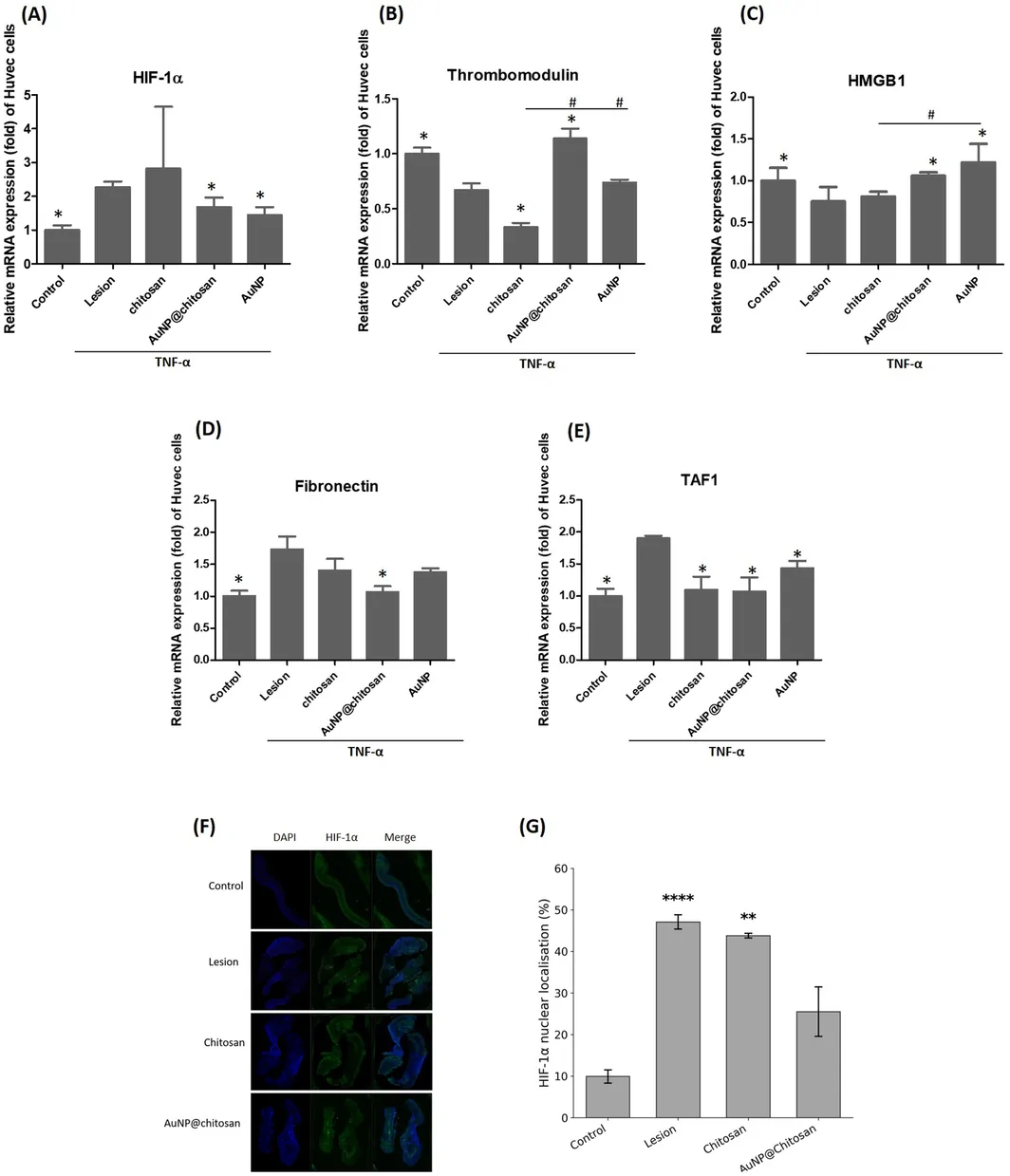
qPCR analysis of hypoxia‐ and thrombosis‐related gene expression in HUVECs and immunofluorescence analysis of HIF‐1α nuclear localization in vivo. (A–E) Expression levels of HIF‐1α, thrombomodulin, HMGB1, fibronectin, and TAF1 were analyzed under TNF‐α stress. AuNP@chitosan moderated HIF‐1α expression toward adaptive levels and downregulated fibronectin and TAF1, reflecting improved cellular adaptation to oxidative stress (*p < 0.05, compared with lesion; ^#^ *p* < 0.05, compared with chitosan). (F–G) Representative Immunofluorescence images and semi‐quantitative analysis of HIF‐1α nuclear accumulation. **p < 0.01 and ****p < 0.0001.

**FIGURE 4:**
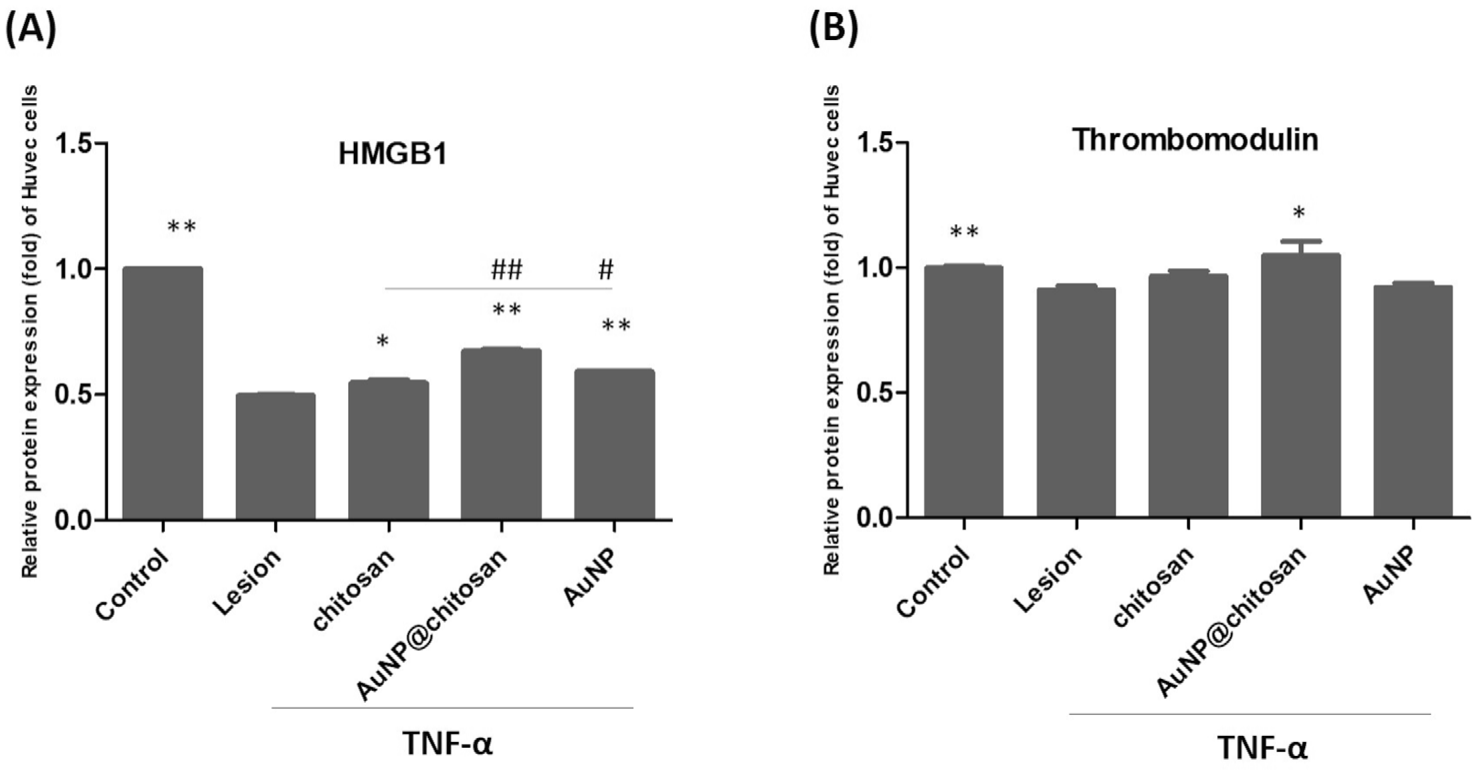
Protein expression of thrombomodulin and HMGB1 in HUVECs after TNF‐α stimulation. ELISA analysis revealed that AuNP@chitosan significantly restored (A) HMGB1 and (B) TM expression levels compared to chitosan alone and TNF‐α control. Data indicate endothelial protection via modulation of coagulation factors (*p < 0.05, **p < 0.01, compared with Lesion; ^#^ *p* < 0.05, ^##^ *p* < 0.01, compared with chitosan).

TM, a key anticoagulant marker on the surface of endothelial cells [12, 18], has significantly decreased in the untreated lesion model compared to the control (~0.75‐fold, mRNA; ~0.91‐fold, protein) (Figures 3 and 4), indicating a mild endothelial injury [19]. AuNP@chitosan has successfully restored TM expression to a level comparable to that of the control (~1.10‐fold, mRNA; ~1.05‐fold, protein), indicating its superior effectiveness compared with sole chitosan treatment in preserving anticoagulant capacity and vascular stability upon TNF‐α stimulation. Although the decreased expression of High Mobility Group Box 1 (HMGB1), which may promote inflammation [17], was restored in the treatment group, its protein levels remained lower than those of non‐injured controls (Figures 3 and 4). Therefore, TNF‐α stimulation did not cause widespread cell necrosis; however, HMGB1 transcription may still be upregulated owing to sublethal stress or early inflammatory signaling (Figure 3). Additionally, HIF‐1α was upregulated more than twofold in the lesion group. Chitosan alone further upregulated HIF‐1α to ~2.8‐fold. The AuNP‐containing treatments alternatively suppressed its expression to 1.5‐ to 1.8‐fold (Figure 3A). Also, HIF‐1α immunofluorescence staining on wound tissue sections showed lower nuclear localization with the AuNP@chitosan group, suggesting that the treatment may attenuate the hypoxia‐associated HIF‐1α response (Figure 3F,G). All treatments also substantially downregulated TAF1, indicating a cytoprotective effect. Similarly, the upregulation of fibronectin, a marker of a pro‐inflammatory state, was mitigated by the treatments, with the AuNP@chitosan formulation showing the most effective downregulation (Figure 3). These results highlight the ability of these materials to restore normal gene expression patterns in injured endothelial cells. Although measured at a single time point, the observed recovery of TM and suppression of HIF‐1α imply that AuNP@chitosan may exert early protective actions.

### 3.4 AuNP@Chitosan Modulates Inflammation in Activated Macrophages

**FIGURE 5:**
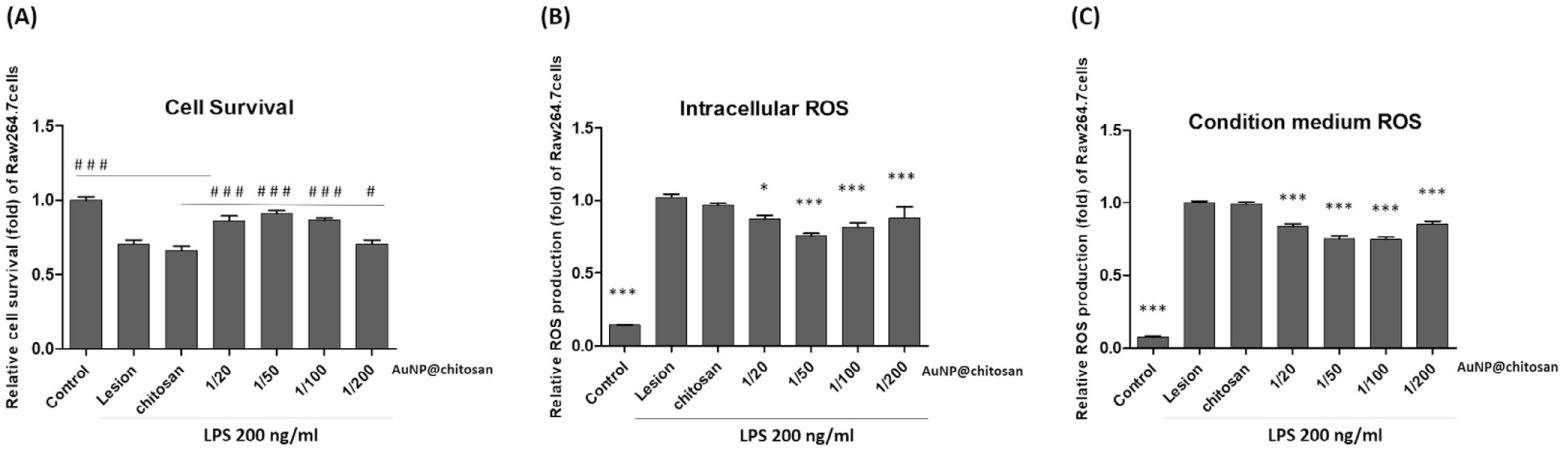
Effect of AuNP@chitosan on ROS levels and cell viability of RAW 264.7 macrophages after LPS stimulation. (A–C) Both intracellular and extracellular ROS were markedly reduced in LPS‐stimulated macrophages at 1/50 and 1/100 dilutions of AuNP@chitosan, confirming that the ROS‐suppressive activity extends to the immune cell compartment in a dose‐dependent manner. Higher dilution groups (1/100 and 1/50) provided significant cytoprotection (*p < 0.05, ***p < 0.001, compared with lesion; ^#^ *p* < 0.05, ^###^ *p* < 0.001, compared with chitosan).

**FIGURE 6:**
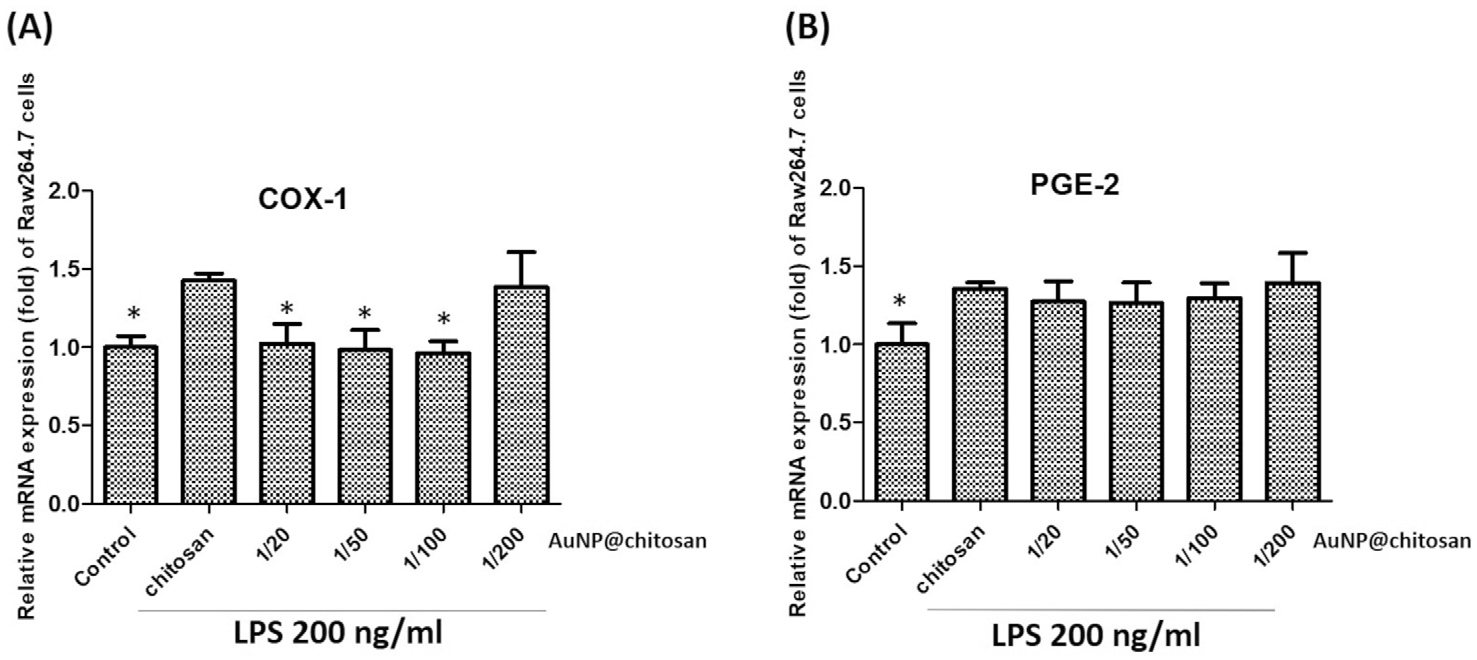
COX‐1 and PGE2 mRNA expression in RAW264.7 macrophages after LPS and material treatment. (A and B) AuNP@chitosan maintained COX‐1 expression at physiological levels while suppressing PGE2, demonstrating a selective COX‐2/PGE2‐targeting profile that spares homeostatic prostaglandin signaling (*p < 0.05, compared with chitosan).

**FIGURE 7:**
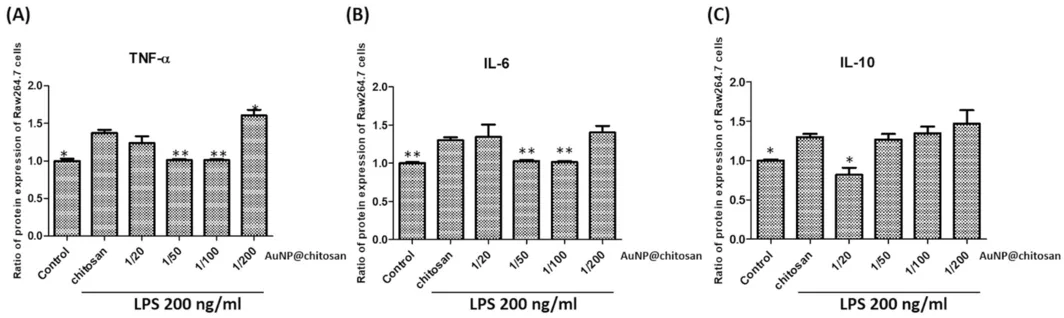
Expression of cytokines TNF‐α, IL‐6, and IL‐10 in RAW264.7 macrophages under LPS stimulation. (A–C) AuNP@chitosan suppressed pro‐inflammatory cytokines (TNF‐α and IL‐6) while enhancing anti‐inflammatory IL‐10 expression, highlighting its immunomodulatory potential (*p < 0.05, **p < 0.01, compared with chitosan).

AuNP@chitosan treatment was assessed in LPS‐stimulated RAW 264.7 macrophages to provide insight into inflammatory cell activation, cytokine expression and secretion, and immune regulatory dynamics. At a 1/20 dilution, AuNP@chitosan exerts moderate effects on cell viability and ROS suppression. Meanwhile, the 1/50 and 1/100 dilutions represented the optimal range, consistently improving cell viability (~0.9‐fold of control), markedly reducing ROS (intracellular and extracellular) (Figure 5), maintaining COX‐1 mRNA levels at a level similar to that of the control, slightly reducing PGE_2_ expression (Figure 6), decreasing pro‐inflammatory cytokines (TNF‐α and IL‐6), and increasing the anti‐inflammatory cytokine IL‐10 (Figure 7). In contrast, the 1/200 dilution showed insufficient effects, with minimal ROS suppression and no improvement in cytokine levels. These results suggest a dose‐dependent property of AuNP@chitosan in exerting an inflammation modulation effect.

### 3.5 AuNP@Chitosan Modulates Immune Cell Infiltration

**FIGURE 8:**
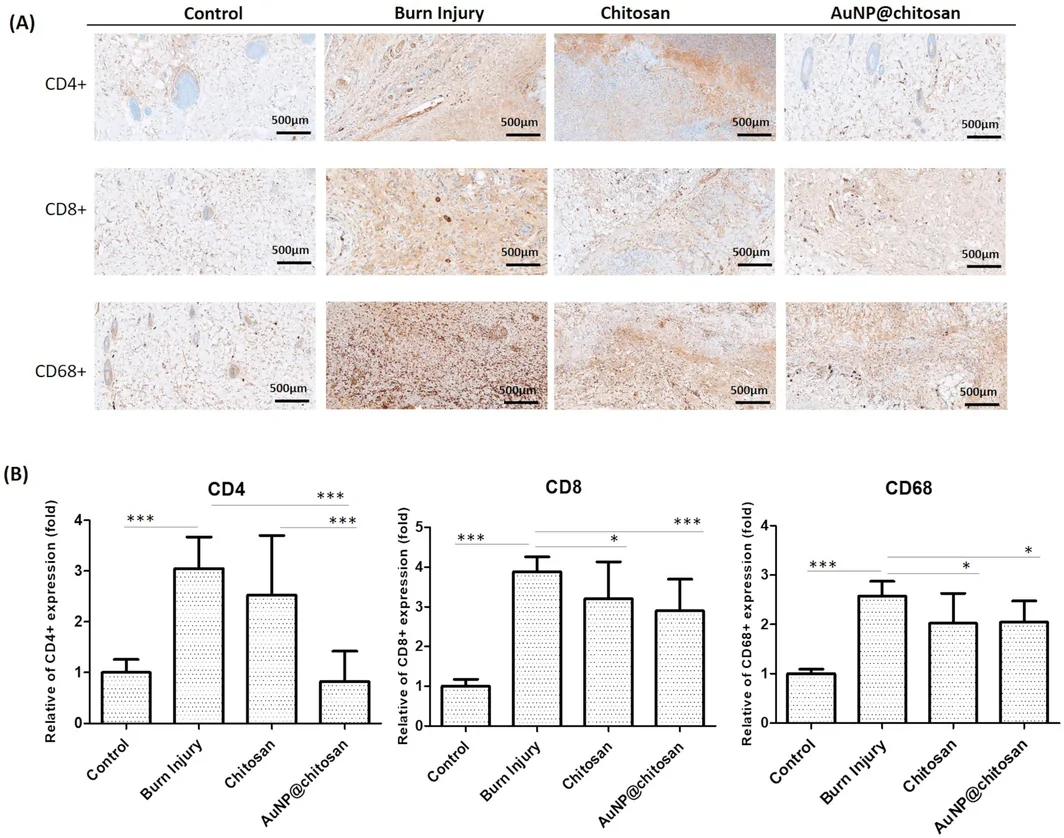
Immunohistochemical analysis of immune cell infiltration in burn‐injured rat skin. (A) Comparison of harvested burned skin tissue through upright microscopy image at 100× magnification. (B) Quantification of CD4^+^, CD8^+^, and CD68^+^ cell populations at Day 7 postburn showed that AuNP@chitosan treatment significantly reduced inflammatory cell infiltration compared with untreated and chitosan‐treated burns (*p < 0.05, ***p < 0.001, compared with chitosan) with TNF‐α at 20 ng.

AuNP@chitosan treatment effectively modulated the immune response in a burn‐injury model by downregulating key immune cell populations (Figure 8). In the burn‐injury group, there was a significant increase in CD4^+^, CD8^+^, and CD68^+^ T‐cell levels (approximately 3‐, 3.8‐, and 2.6‐fold, respectively). AuNP@chitosan treatment effectively mitigated this response, significantly downregulating CD4^+^ T‐cell levels to a range comparable to that of the control group (*p* < 0.001). This reduction indicates the attenuation of excessive immune activation, which favors a balanced inflammatory response crucial for establishing the immunological balance required for subsequent tissue repair. Similarly, AuNP@chitosan relieved the intense cytotoxic immune reaction by significantly suppressing CD8^+^ T‐cell levels (*p* < 0.001 vs. burn injury; *p <* 0.05 vs. chitosan group), suggesting that the treatment can modulate oxidative stress and prevent further collateral damage. The increase in CD68^+^ expression after burn injury, which reflects increased macrophage infiltration and inflammation, was also significantly reduced by AuNP@chitosan (*p* < 0.05), with the combination treatment providing superior suppression compared to chitosan alone. These findings suggest that AuNP@chitosan effectively regulates macrophage‐driven inflammatory responses during wound healing.

## 4 Discussion

The data collectively demonstrate that the AuNP@chitosan composite acts as a powerful, multi‐target therapeutic agent for mitigating trauma and burn‐induced pathology, exhibiting superior efficacy over chitosan alone across both endothelial and immune cell models, which are critical to the wound ecosystem. The integrity of the vascular endothelium, represented by HUVECs, is central to this pathology, where the key anticoagulant marker, TM, is compromised by inflammation. We confirmed that TNF‐α‐induced oxidative stress (ROS) drives endothelial dysfunction, thereby suppressing TM and promoting a procoagulant, inflammatory phenotype [2, 20, 21]. Building on prior work suggesting AuNPs influence the coagulation system [22], AuNP@chitosan effectively countered this damage: it significantly reduced ROS levels (Figure 2) [23] while restoring both TM protein and mRNA expression (Figures 3 and 4) to levels comparable to the non‐lesion state (Figures 3 and 4). This rapid ROS suppression is likely driven by a direct‐scavenging mechanism via the dithiol groups of the DHLA surface ligands and the hydrogen‐donor amine groups of chitosan—a chemical process that quenches reactive species independently of transcriptional Nrf2/HO‐1 upregulation [24]—though the secondary contribution of endogenous antioxidant pathways cannot be entirely excluded at this stage and remains a valuable avenue for future investigation. This restoration suggests a functional benefit in preserving anticoagulant capacity and vascular stability. Crucially, the increased TM levels after treatment may further neutralize HMGB1 and prevent its pro‐inflammatory signaling (e.g., TLR4 and RAGE) [25]. The fact that TM and HMGB1 protein levels were elevated (Figure 4) and returned closer to the non‐lesion state (Figures 3 and 4) with statistical significance (*p* < 0.05 compared to the lesion group) indicates the treatment's strong potential to restore balance in the wound ecosystem. Furthermore, the composite supports cellular adaptation, indicated by the regulation of HIF‐1α, fibronectin, and TAF‐1 (Figure 3), through enhancing endothelial survival and antithrombotic capacity in hypoxic conditions by promoting angiogenesis, contributing to vascular integrity and repair [26]. The concurrent modulation of HIF‐1α expression and restoration of TM observed in Figure 3 merits particular attention as a mechanistically integrated anticoagulant–immunoprotective response. Under TNF‐α–driven inflammatory injury, HIF‐1α is stabilized through NF‐κB‐dependent, non‐hypoxic mechanisms; its moderate expression (1.5‐ to 1.8‐fold) in AuNP@chitosan‐treated HUVECs—compared to the excessive induction in the untreated lesion group (> 2‐fold)—reflects a shift from a pathological to an adaptive, cytoprotective expression level. This distinction is critical. HIF‐1α is an essential regulator of myeloid cell‐mediated inflammatory responses, including macrophage survival and bactericidal function under inflammatory stress, underscoring that HIF‐1α acts as a central coordinator of innate immunity far beyond its canonical hypoxia‐sensing role [27]. HIF‐1α was further demonstrated to regulate phagocyte bactericidal capacity through antimicrobial peptide production and granule formation [28]. From the endothelial perspective, hypoxia‐inducible pathways are intimately coupled to vascular inflammatory and coagulant signaling, with HIF‐1α modulating endothelial barrier function and coagulant state [29]. The simultaneous recovery of TM—the endothelial surface anticoagulant that catalyzes protein C activation, thereby dampening both coagulation and inflammation—alongside HIF‐1α modulation (Figure 3) therefore reflects a coherent anticoagulant–immune reprogramming of the injured endothelium. This HIF‐1α/TM co‐regulatory axis, confirmed in Figure 3, is a mechanistically specific outcome that distinguishes AuNP@chitosan from nonselective anti‐inflammatory agents and from all prior AuNP@chitosan composites, none of which have reported TM restoration as a functional endpoint. While further characterization of canonical nuclear translocation kinetics remains an informative avenue for future study, our primary focus here rests on this coordinated gene expression profile as the functional readout of the inflammatory coagulation‐immune interface.

This observed endothelial protection is complemented by robust immunomodulatory effects on inflammatory cells, as demonstrated in LPS‐stimulated RAW 264.7 macrophages. While chitosan possesses intrinsic immunomodulatory properties [6], AuNP@chitosan exhibited a more pronounced anti‐inflammatory action by suppressing the typical NF‐κB signaling pathway, which led to upregulation of pro‐inflammatory mediators [4], as shown by the composite's ability to significantly reduce TNF‐α and IL‐6, while simultaneously promoting the anti‐inflammatory cytokine IL‐10 (Figures 6 and 7). Unlike chitosan alone, which showed signs of mild immune activation (Figure 7), AuNP@chitosan achieved this anti‐inflammatory state by reducing ROS generation and likely attenuating TLR4‐mediated signaling [30]. It is worth noting that the mechanism described here differs from the antibacterial paradigm of silver‐based standards, such as silver sulfadiazine [31]. While silver is indispensable for its potent bactericidal effects, our composite is designed to specifically interface with host inflammatory responses. The cytokine modulation profile of a commercial silver‐containing hydrofiber dressing (AQUACEL Ag) was evaluated (Figures S1 and S2). The silver‐based dressing demonstrated an expected fluctuation in pro‐inflammatory markers (TNF‐α and IL‐6) over a 7‐day period—primarily as a secondary effect of its bactericidal activity—and these data serve as a reference point for the distinct, host‐targeted immunomodulatory mechanism observed with our AuNP@chitosan composite, which may offer a nuanced approach to stabilizing the wound ecosystem by mitigating early‐stage endothelial and immunological hyperactivation. Critically, the composite also affects prostaglandin signaling in a highly selective manner: it profoundly inhibits PGE_2_ protein production (also slightly at the mRNA level) and downregulates the COX‐2/PGE_2_ axis while maintaining COX‐1 expression within physiological ranges (Figure 6). This selective modulation is crucial for preventing the disruption of essential homeostatic prostaglandin signaling [32]. This COX‐1‐sparing, PGE_2_‐suppressive pharmacological profile is analogous to that of COX‐2‐selective NSAIDs but achieved through a nanomaterial‐mediated mechanism and without the associated cardiovascular risk concerns. Critically, this selectivity—preserving COX‐1‐dependent homeostatic prostaglandin synthesis essential for gastric mucosal integrity, platelet function, and vascular tone while specifically targeting pathological PGE_2_ overproduction in activated macrophages—has not been previously documented for any AuNP@chitosan composite and constitutes a more refined immunomodulatory approach than broad anti‐inflammatory interventions. Notably, AuNP@chitosan exhibits these benefits at intermediate concentrations, revealing a dose‐dependent modulation pattern to provide optimal balance.

Complex injuries such as burns involve a cascade of events from endothelial damage to aggressive immune cell infiltration and macrophage activation; therefore, the significance of AuNP@chitosan as a wound dressing should not be evaluated in the context of a single cell type. By employing two distinct models, HUVECs for endothelial function and RAW 264.7 macrophages for immune regulation, we provided mechanistic insights that translate to the physiological context of a burn animal model. The in vitro ability of AuNP@chitosan to suppress PGE_2_ and downregulate inflammatory markers (Figure 7) correlates with its in vivo finding of significantly reduced infiltration of CD4^+^, CD8^+^, and CD68^+^ immune cells in the burn wound (Figure 8). This suggests the composite effectively mitigates excessive immune cell recruitment and maintains immunological balance during tissue repair. Collectively, the combined cellular and animal data demonstrate that AuNP@chitosan establishes a pro‐healing immunological environment through a multipronged mechanism: direct attenuation of oxidative stress (ROS), suppression of NF‐κB‐mediated cytokine overproduction, and resolution of pathological immune cell infiltration. These mechanistic effects—operating at the coagulation–immune interface—constitute the necessary vascular and immunological preconditions for subsequent tissue repair. While this study focuses on these essential early‐stage milestones, subsequent investigations involving extended time points, collagen deposition analysis (e.g., Masson's trichrome staining), and detailed angiogenesis markers are warranted to fully characterize the transition from this pro‐healing environment to complete reepithelialization and tissue remodeling.

## 5 Conclusions

This study demonstrates that chitosan conjugated with AuNPs (AuNP@chitosan) effectively restores endothelial TM expression, reduces oxidative stress, and selectively modulates inflammatory mediators in both endothelial and macrophage cell models. Compared to chitosan alone, AuNP@chitosan significantly suppresses ROS and pro‐inflammatory cytokines (TNF‐α, IL‐6, and PGE_2_) while maintaining COX‐1 and enhancing IL‐10 expression. These dual regulatory effects were confirmed in a burn wound rat model, where AuNP@chitosan reduced immune cell infiltration (CD4^+^, CD8^+^, and CD68^+^). These findings suggest that AuNP@chitosan improves wound healing by targeting both oxidative and immune dysregulation through a biomaterial–cell interface mechanism.

## Author Contributions

**Juin‐Hong Cherng:** conceptualization, formal analysis, investigation, resources, supervision, validation. **Sheng‐Der Hsu:** conceptualization, funding acquisition, project administration, resources, software, validation. **Gang‐Yi Fan:** data curation, investigation, methodology, visualization. **Hsin‐Da Tsai:** data curation, investigation, methodology, visualization. **Amber Lin:** investigation. **Cheng‐An Lin:** resources, validation. **Shu‐Jen Chang:** validation, writing – original draft, writing – review and editing.

## Funding

This work was supported by the National Defense Medical Affairs Bureau (MND‐MAB‐D‐112‐0035, MND‐MAB‐D‐114‐159, MND‐MAB‐D‐114119, MND‐113‐0048, MND‐114‐0075), the National Defense Medical University and Chung Yuan Christian University cooperation grant (111/112/113/114), the Tri‐Service General Hospital (TSGH_D_114127), the National Defense Development Foundation (grant number 020112002), and the National Defense Medical University (NDMU‐HESP120103).

## Conflicts of Interest

The authors declare no conflicts of interest.

## Supporting information


**Figure S1:** Serum inflammatory cytokine profiles in a rat burn model treated with standard gauze (untreated control), calcium alginate dressing, or commercial silver‐containing hydrofiber dressing (AQUACEL Ag). (A) IL‐6 and (B) TNF‐α concentrations (pg/mL) were measured at pre‐burn baseline (Day 0) and post‐dressing Days 1, 3, 7, 14, and 28 using a multiplex bead‐based immunoassay (MILLIPLEX MAP, Merck KGaA). The treatment groups included gauze, calcium alginate, and AQUACEL Ag. A pronounced late‐phase resurgence of IL‐6 was observed in the AQUACEL Ag group at Day 28. Error bars represent SD (*n* = 2 biological replicates per time point for calcium alginate and AQUACEL Ag groups; gauze group represents individual measurements).

**Figure S2:** Serum cyto‐/chemokine profiles in a porcine contact thermal injury model treated with calcium alginate dressing (CAPS) or commercial silver‐containing hydrofiber dressing (AQUACEL Ag). Concentrations of (A) IL‐4, (B) IL‐6, (C) IFN‐γ, (D) MCP‐1, (E) TNF‐α, (F) TGF‐β1, (G) TGF‐β2, and (H) TGF‐β3 were measured in serum collected at postburn Days 0, 1, and 7 using a multiplex bead‐based immunoassay (MILLIPLEX MAP, Merck KGaA). AQUACEL Ag was associated with significantly elevated IFN‐γ (Day 7) and MCP‐1 (Day 1) compared to CAPS. In contrast, CAPS demonstrated significantly lower levels of the wound‐remodeling cytokines TGF‐β1 (Days 1 and 7), TGF‐β2 (Day 7), and TGF‐β3 (Day 1) compared with AQUACEL Ag. Values represent mean ± SD (*n* = 5 per group). *p < 0.05, **p < 0.01, and ***p < 0.001 between groups at each time point by Student's *t*‐test.

## Data Availability

All data generated or analyzed in this study are included in this article and its Supporting Information files. Additional raw data are available from the corresponding author upon reasonable request.
